# Raters and examinees training for objective structured clinical examination: comparing the effectiveness of three instructional methodologies

**DOI:** 10.1186/s12912-024-02183-6

**Published:** 2024-07-23

**Authors:** Jefferson Garcia Guerrero, Ayidah Sanad Alqarni, Lorraine Turiano Estadilla, Lizy Sonia Benjamin, Vanitha Innocent Rani

**Affiliations:** College of Nursing, King Khalid University, Abha Al-Qureiger Campus, Abha, Aseer Region, 62529 Saudi Arabia

**Keywords:** Instructional methodology, Simulation-based learning, Traditional lectures, Online instructions, Objective structured clinical examination

## Abstract

**Background:**

Utilizing the objective structured clinical examination (OSCE) ensures objectivity when it comes to the assessment of nursing students’ skills and competency. However, one challenge in OSCE integration is rater and examinee training and orientation.

**Aim:**

This study employed a quasi-experimental design to evaluate the effectiveness of different instructional methodologies in training and preparing raters and examinees for the OSCE.

**Methods:**

Participants were divided into three group of training methodologies: online, simulation, and traditional lecture (six raters and 18 examinees were assigned to each group). A total of 18 raters and 54 examinees partook.

**Results:**

The study found that raters trained through simulation exhibited a slight agreement with their rates, compared to those who were trained online and in traditional lectures. Moreover, examinees who were trained through the simulation methodology performed better compared to those trained via the other methodologies.

**Conclusions:**

The study findings indicate that using simulation by training raters and examinees in the OSCE is the most effective approach.

**Supplementary Information:**

The online version contains supplementary material available at 10.1186/s12912-024-02183-6.

## Introduction

The objective structured clinical examination (OSCE) is a comprehensive and standardized objective instrument for evaluating clinical skills and competencies, as well as an effective teaching strategy [1]. The standardized format of the OSCE ensures a consistent and reliable evaluation of specific nursing skills, enabling an accurate assessment of student progress [2]. Furthermore, the structured nature of the OSCE enables educators to provide immediate and targeted feedback, empowering students to refine their skills and build confidence [3, 4]. However, past work also found that OSCE raters with less experience were more likely to judge compared to those raters with a wealth of experience [5].

Using scenarios such as medication administration or patient communication, the OSCE assesses the competency of nursing students in a real-life context, helping them prepare for future clinical practice [3, 4]. Extensive research supports the effectiveness of the OSCE as a clinical competency assessment tool. Its high validity and reliability make it the gold standard for accurate and objective evaluations [6]. However, the students must be able to undergo appropriate OSCE training and preparation first to ensure that they are familiar with the process [7]. Additionally, all assessment methods have flaws, limitations, and shortcomings, meaning it is important to be aware of the rater’s perception and student’s performance [5].

Beyond its assessment capabilities, the OSCE demonstrably motivates students to learn, strengthens their communication skills, and hones their basic nursing proficiency. This fosters confidence in their clinical abilities and overall satisfaction with their learning journey [8, 9]. Participation in the OSCE elicited positive emotions in students who appreciated the simulated realism, constructive feedback, and opportunities for skill development [9]. This and its perceived value as a learning method reinforce its positive impact on nursing education [9, 10]. Studies have shown that OSCE performance scores are highly correlated with the subsequent performances of students in clinical practice. Its predictive value makes it a crucial tool for evaluating students’ professional practice readiness [11]. Despite the reported positive effects of the OSCE, there are several challenges linked with its organisation and implementation, such as inadequate training and orientation of raters and examinees, poorly designed scenarios, lack of skills laboratory and equipment needed for the scenarios, inadequate number of OSCE stations (potentially meaning that not all learned competencies are represented), and inadequate time allocation on each station, thus possibly affecting the performance of both raters and examinees, including the validity and reliability of the OSCE [12]. Additionally, raters are also prone to exhaustion, which could lead to unfair evaluation in a large number of examinees [13]. Furthermore, proper training and preparation for the OSCE is important when it comes to achieving a positive result. The Adult Learning Theory, by Malcolm Knowles (1968), acknowledged that the learning acquired by adults is more effective when such adults use their skills, experience, and knowledge. Introducing new methodologies of teaching requires the integration of the principles regarding how to encourage participants to learn, and the OSCE appears to be a perfect example [14].

Moreover, adequate preparation time for students significantly optimizes their OSCE performance. Additionally, Yusuf [6] emphasises the benefits of implementing mock OSCEs. These simulated assessments offer valuable practical opportunities, allowing students to familiarize themselves with the OSCE format, receive constructive feedback, and build confidence before an actual exam [15]. However, Hyde et al. [5] reported that no assessment method exists without any inherent limitations. Consequently, carefully considering these limitations and their potential influence on student performance and examiner perception is essential for effective assessment implementation. Additionally, evidence regarding the effectiveness of rater training in boosting scoring reliability and accuracy in OSCE settings is limited.

Although it has been reported that the OSCE is an effective strategy in assessing students’ knowledge, clinical competency [9], critical thinking, and problem-solving skills [16], the tool has only been utilised to a limited extent in nursing education [17]. OSCE-associated glitches include shortage of trained OSCE raters [9, 18], absence of training for raters, rater’s intimidation, OSCE stations’ time shortage, and increased stress amongst students [11, 19]. Ataro et al. [11] also reported that many students who experienced the OSCE complained of lack of training and preparation time before the examination. Additionally, the evidence on safeguarding the validity, reliability, and internal consistency of OSCE checklists remains inadequate [20]. Therefore, appropriate training and preparation of raters and examinees for the OSCE is vital when it comes to ensuring positive outcomes and providing a meaningful learning experience.

Traditional lectures have long been the dominant strategy in healthcare education [21]. This strategy is effective in delivering core knowledge and explaining concepts that are difficult to understand, thereby enhancing learning. Conventional lectures encourage engagement and self-directed learning. Afrasiabifar and Asadolah [22] provided further evidence for the effectiveness of interactive lectures in nursing education, highlighting their ability to improve learning outcomes and promote active student engagement in the teaching–learning process.

Furthermore, information technology has been integrated into nursing education, making computers indispensable platforms for teaching and learning. Technology is readily accessible in the digital marketplace, connecting teachers and learners worldwide and allowing them to engage in vibrant dialogue [23].

Simulation-based learning (SBL) offers an immersive environment that is effective and powerful when it comes to the progress of learners [24]. SBL acts as a crucial bridge, seamlessly transporting knowledge gleaned from clinical training to the real world of nursing practice [25], and offers a safe environment for experimentation and learning. Students can practice in challenging situations, make errors without harming the patient, and receive immediate feedback to refine their approaches. This fosters a fearless learning environment in which curiosity and critical thinking flourish [4]. Additionally, simulation training is an effective and efficient strategy for advancing professional nurse’s competence [26].

Traditional methods for assessing nursing skills, performance, and competency are often used in the Bachelor of Science in Nursing (BSN) programme, although they are commonly not structured or objective. Therefore, the Quality Development and Academic Accreditation Unit of the College of Nursing at King Khalid University offers a BSN programme aimed at integrating the best practices of OSCE to ensure the objectivity of student skill and competency assessments. However, one challenge in OSCE integration is the preparation and orientation of faculty members and students. The lack of OSCE rater training leads to poor organisation of the assessment process [11], and raters feel a lack of confidence in rating during assessment [5].

Integrating the OSCE into the assessment of the clinical skills, performance, and competency of nursing students in the BSN programme at the College of Nursing at King Khalid University is a milestone. The OSCE offers valuable information on the strengths and weaknesses of students; it encourages self-awareness of their professional competencies. Therefore, this study aims to evaluate the effectiveness of different instructional methodologies, such as online instructions, simulations, and traditional lectures, in training and preparing raters and examiners for the OSCE.

## Methods

### Design

This study employed a quasi-experimental research design to evaluate the effectiveness of different instructional methodologies, such as online instructions, simulations, and traditional lectures, in training and preparing raters and examiners for the OSCE.

### Participants

The participants were selected based on the following criteria: raters must be nurses with a master’s degree and a doctorate degree, a full-time faculty member of the College of Nursing at the King Khalid University for at least one year, and without prior experience of conducting the OSCE; the examinees must be nursing students who are officially enrolled in the BSN programme at the College of Nursing of King Khalid University, having completed the Fundamentals of Nursing I and II courses, with a general point average (GPA) of 3.5 and above out of 5 for the last two years, and without OSCE experience.

Additionally, two well-trained OSCE specialists were invited to conduct the training: one medical doctor with a master’s and doctorate degree in medical education, and a nurse with a master’s and doctorate degree in nursing. The two well-trained OSCE specialists who conducted the training also served as the evaluators. The rater and examinee groups were trained using three approaches: traditional lectures, remote instructions, and simulations. The trainers also acted as organizers and OSCE timekeepers. Furthermore, this study was conducted in December 2023 at the King Khalid University, College of Nursing, Abha.

### Interventions

The data collection was conducted for three consecutive days, with the topics of the training focused on the organization and implementation of the OSCE. On day one, the first rater and examinee groups were trained through traditional face-to-face lectures. The lecture was conducted in one of the university auditoria using a PowerPoint presentation, with the discussion lasting 1 h and 20 min. Subsequently, all questions from the raters and examinees were addressed—a process which took 30 min.

On day two, the second group was trained remotely using online conferencing. The discussion was conducted through the Blackboard Collaborate platform using the same PowerPoint presentation employed during the face-to-face lecture. The discussion lasted 1 h and 10 min. Subsequently, all questions from the raters and examinees were addressed, with this process lasting 25 min.

On day three, the final group was trained using a simulated OSCE; this group underwent pre-briefing, actual OSCE, and debriefing. During the pre-briefing, the trainers provided a brief orientation to the raters and examinees regarding their roles and how the OSCE is conducted, such as the time and how they transfer from one station to another. The trainers also discussed how the students have only 6 min at every station—1 min to read and analyse the scenario and 5 min to perform the task. The pre-briefing lasted for 35 min, with the actual OSCE lasting for 1 h and 48 min, followed by the 40-minute-long debriefing. All forms of training (online, traditional, and simulation) were conducted by the two well-trained OSCE specialists.

There were six OSCE station sets, each with an equivalency of 10%, and the average was calculated to obtain the total OSCE score of each student. Laboratory rooms were prepared with all the equipment required for the procedure, and scenarios were posted outside the OSCE station rooms. The organiser ensured that the students were confined to the classroom; they were permitted to leave only when asked to take the examination in the laboratory. Mobile phones and other types of communication technology were prohibited in the classrooms. During the OSCE, the examinees were given 1 min to read and analyse the scenario before entering the rooms. When the examinee entered the room, their time of 5 min started. Debriefing was conducted after the OSCE. All OSCEs were carried out immediately after each training session had been conducted. The feedback regarding the experiences of raters and examinees with the OSCE training was collected immediately after the OSCE debriefing, and the trainers’ observation checklists were completed by the trainers at the same time.

Additionally, an observational survey consisting of six indicators was conducted by the trainers for each training approach. The raters and examiners also performed self-assessments regarding their OSCE training experience.

### Research instruments

The researchers prepared six scenarios and rubrics related to basic nursing procedures: (1) intradermal injection, (2) intramuscular injection, (3) nasogastric tube insertion, (4) urinary catheterisation in male patients, (5) urinary catheterisation in female patients, and (6) blood pressure measurements. The nursing procedural rubrics used in the present study were adopted from Taylor’s clinical nursing skills [27]. All rubrics used a 4-point Likert scale: 3 (performed correctly), 2 (performed incompletely), 1 (performed incorrectly), and 0 (not performed), depending on the procedure. The total score for each station was 10 points, and the final score was calculated by averaging all the OSCE stations. To ensure the reliability of the rubrics, 18 preceptors were invited to take a mock OSCE before the study. The six OSCE stations from one to six had reliability scores of 0.91, 0.90, 0.86, 0.88, 0.91, and 0.87 for the kappa coefficients, respectively. A kappa result of 0.81–1.00 indicated almost perfect agreement [28].

Additionally, the researchers developed three surveys to evaluate feedback regarding the experiences of raters and examinees with the OSCE training, including the observations of raters [the English language version of the survey is attached as a supplementary file]. A trainer’s observation checklist, consisting of six indicators, was used to evaluate the overall conduct of the OSCE training. Further, the raters and examiners took self-assessment surveys on their OSCE training experience, with each survey comprising five indicators. All surveys employed a 3-point Likert-type scale: 3 (agree), 2 (partially agree), and 1 (disagree). A reliability of 0.81 on the OSCE trainer’s observation checklist, 0.81 on the rater’s perception of the OSCE training survey, and 0.81 on the examinee’s perception of the OSCE training survey were recorded using McDonald’s Omega. An internal consistency value of more than 0.70 indicated acceptability [29].

### Sample size

The sample size for each group (online instruction, simulation, and traditional lecture) was calculated based on a G*Power calculation with a 5% margin of error, 95% confidence level, and 80% power of the test.

### Randomisation

The participants were randomly allocated using their employee and student numbers via a paper lottery system. The respondents comprised three faculty member groups, with six members in each group, who functioned as raters, and three student groups, with 18 members in each group, who acted as examinees. We selected 72 participants (18 raters and 54 examinees). Subsequently, six raters and 18 examinees each were allocated to groups A (raters and examinees trained in OSCE through traditional lectures), B (remote instructions), and C (simulation).

### Statistical methods

Data were analysed using Stata version 17. McDonald’s ω and Cronbach’s α were utilised to assess the frequentist scale reliability statistics of the tools used (“OSCE Training Observation Checklist”, “Student’s Perception on OSCE Training”, and “Rater’s Perception on OSCE Training”). Cohen’s unweighted kappa and Fleiss’ kappa were used to assess the rater agreement reliability. The demographic data of the raters and the performance of the examinees were analysed using the frequency and percentage of the count data. Means and standard deviations (SD) were calculated for each continuous variable to assess the perceptions of the raters, examinees, and trainers regarding the three instructional methodologies. A single-factor analysis of variance was used to compare the effectiveness of the three instructional methodologies (online, simulation, and traditional lectures). Finally, a post hoc analysis was employed to assess the differences amongst multiple groups whilst avoiding experiment-wise errors.

The final OSCE scores of the examinees were interpreted as 0–2 (very poor), 3–5 (poor), 6–8 (good), or 9–10 (very good). The effectivity of the instructional methodologies based on the perceptions of the raters, examiners, and trainers was scored as follows: 1–1.49 (not effective), 1.50–2.49 (slightly effective), and 2.50–3.00 (effective). Cohen’s kappa and Fleiss’ kappa values were interpreted according to the Landis and Koch (1977) guidelines of agreement level [30]: less than 0 (poor), 0.0–0.2 (slight), 0.21–0.40 (fair), 0.41–0.60 (moderate), 0.61–0.80 (substantial), and 0.81–1.0 (almost perfect or perfect).

### Ethical considerations

We received ethical approval from the King Khalid University Research Ethics Committee (approval no. ECM#2023 − 1601). Each participant provided written informed consent. The researcher gave a detailed explanation of the purpose of the study, emphasising the participants’ right to withdraw at any point. Strict measures were implemented to protect the privacy of the participants and ensure the confidentiality of all collected information.

## Results

Table 1 shows the demographic profile of the OSCE raters and examinees. The majority of the OSCE raters were female (88.88%), aged 36–45 years (33.33%) and 46–55 years (33.33%), with an academic rank of assistant professor (61.11%) and Egyptians by nationality (33.33%). The examinees were all female nursing students, Saudi nationals, aged 20–21 years (44.44%), and currently in the 3rd year of the BSN programme (51.85%).

**Table 1:** Demographic profile of the OSCE raters and examinees

Demographic Profile	Frequency	Percentage
OSCE Raters		
Gender		
Male	2	11.11
Female	16	88.88
***Age***		
25–35 years	5	27.77
36–45 years	6	33.33
46–55 years	6	33.33
56 years and above	1	5.55
***Educational qualification***		
Master’s degree	7	38.88
Doctorate degree	11	61.11
***Nationality***		
Egyptian	6	33.33
Filipino	3	16.66
Indian	3	16.66
Saudi	4	22.22
Sudanese	2	11.11
***OSCE Examinees***		
***Gender***		
Male	0	0.00
Female	18	100
***Age***		
20–21 years	24	44.44
22–23 years	23	42.59
24–25 years	7	12.96
***Year level***		
3rd year	28	51.85
4th year	26	48.15

Table 2 presents the rater’s agreement reliability on online methodology used in OSCE training. The highlighted results of Cohen’s unweighted kappa (rater 1 – rater 2 = -0.070, rater 1 – rater 3 = -0.042, rater 2 – rater 3 = -0.047, rater 1 – rater 4 = -0.138, rater 2 – rater 4 = -0.047, rater 1 – rater 5 = -0.024, rater 3 – rater 5 = -0.016, rater 4 – rater 5 = -0.067, rater 1 – rater 6 = -0.043, rater 3 – rater 6 = -0.040, and rater 4 – rater 6 = -0.033) indicate that these raters have a poor level of agreement. The Cohen’s average kappa of -0.021 also indicates a poor level of agreement amongst the raters.

**Table 2:** Raters’ agreement reliability on online methodology used in OSCE training

Cohen’s Unweighted Kappa				
			**95% CI**	
**Ratings**	**Unweighted Kappa**	**SE**	**Lower**	**Upper**
Average Kappa	**-0.021**			
Rater 1 - Rater 2	**-0.070**	0.059	-0.185	0.045
Rater 1 - Rater 3	**-0.042**	0.029	-0.099	0.016
Rater 2 - Rater 3	**-0.047**	0.102	-0.246	0.153
Rater 1 - Rater 4	**-0.138**	0.104	-0.343	0.066
Rater 2 - Rater 4	**-0.047**	0.109	-0.261	0.168
Rater 3 - Rater 4	0.110	0.091	-0.069	0.288
Rater 1 - Rater 5	**-0.024**	0.103	-0.227	0.178
Rater 2 - Rater 5	0.091	0.070	-0.047	0.229
Rater 3 - Rater 5	**-0.016**	0.016	-0.047	0.015
Rater 4 - Rater 5	**-0.067**	0.077	-0.218	0.085
Rater 1 - Rater 6	**-0.043**	0.064	-0.168	0.081
Rater 2 - Rater 6	0.012	0.121	-0.226	0.249
Rater 3 - Rater 6	**-0.040**	0.083	-0.203	0.124
Rater 4 - Rater 6	**-0.033**	0.127	-0.281	0.215
Rater 5 - Rater 6	0.032	0.083	-0.131	0.196
***Fleiss’ Kappa***				
			**95% CI**	
**Ratings**	**Fleiss’ Kappa**	**SE**	**Lower**	**Upper**
Overall	**-0.044**	0.027	-0.097	0.009
2	**-0.038**	0.061	-0.157	0.081
3	**-0.026**	0.061	-0.145	0.093
4	**-0.080**	0.061	-0.199	0.039
5	**-0.017**	0.061	-0.136	0.102
6	**-0.076**	0.061	-0.195	0.043
7	**-0.075**	0.061	-0.194	0.044
8	0.055	0.061	-0.064	0.174
9	**-0.019**	0.061	-0.138	0.100

Furthermore, the total agreement of the raters was poor, as suggested by the overall Fleiss’ kappa of -0.044. The Fleiss’ kappa values were also included in the rating system from 2 to 9 out of 10. Additionally, the aforementioned reflects that the raters have poor agreement between ratings of 2, 3, 4, 5, 6, 7, and 9, with Fleiss’ kappa values of -0.038, -0.026, -0.080, -0.017, -0.076, -0.075, and − 0.019, respectively (see Table 1). According to Landis and Koch [30], a Cohen’s kappa and Fleiss’ kappa less than 0 indicates poor agreement.

Table 3 illustrates the rater’s agreement reliability on simulation methodology used in OSCE training. The highlighted results of the Cohen’s unweighted kappa (rater 2 – rater 4 = -0.033 and rater 3 – rater 4 = -0.268) indicate that these raters have a poor level of agreement. However, the Cohen’s average kappa of 0.196 indicates slight agreement amongst the raters.

**Table 3:** Raters’ agreement reliability on simulation methodology used in OSCE training

Cohen’s Unweighted Kappa				
			**95% CI**	
**Ratings**	**Unweighted Kappa**	**SE**	**Lower**	**Upper**
Average Kappa	0.196			
Rater 1 - Rater 2	0.397	0.167	0.070	0.724
Rater 1 - Rater 3	0.260	0.155	-0.043	0.563
Rater 2 - Rater 3	0.397	0.166	0.072	0.722
Rater 1 - Rater 4	0.075	0.177	-0.272	0.422
Rater 2 - Rater 4	**-0.033**	0.187	-0.400	0.333
Rater 3 - Rater 4	**-0.268**	0.138	-0.538	0.002
Rater 1 - Rater 5	0.053	0.135	-0.212	0.317
Rater 2 - Rater 5	0.339	0.173	-3.755e-4	0.679
Rater 3 - Rater 5	0.333	0.161	0.018	0.649
Rater 4 - Rater 5	0.189	0.188	-0.179	0.557
Rater 1 - Rater 6	0.167	0.171	-0.169	0.502
Rater 2 - Rater 6	0.571	0.148	0.282	0.861
Rater 3 - Rater 6	0.339	0.166	0.014	0.665
Rater 4 - Rater 6	0.000	0.148	-0.290	0.290
Rater 5 - Rater 6	0.116	0.159	-0.196	0.428
***Fleiss’ Kappa***				
			**95% CI**	
**Ratings**	**Fleiss’ Kappa**	**SE**	**Lower**	**Upper**
Overall	0.191	0.040	0.113	0.270
6	**-0.009**	0.061	-0.128	0.110
7	0.224	0.061	0.105	0.343
8	0.173	0.061	0.054	0.292
9	0.111	0.061	-0.008	0.230
10	0.296	0.061	0.177	0.415

Furthermore, the total agreement of the raters was in accord, as suggested by the overall Fleiss’ kappa of 0.191. The Fleiss’ kappa values were also included in the rating system from 6 to 10 out of 10. Additionally, this reflects that the raters have poor agreement on the rating of 6, with a Fleiss’ kappa value of -0.009 (see Table 2). According to Landis and Koch [30], a Cohen’s kappa and Fleiss’ kappa result less than 0 indicates poor agreement.

Table 4 shows the rater’s agreement reliability on online methodology used in OSCE training. The highlighted results of Cohen’s unweighted kappa (rater 1 – rater 3 = -0.146, rater 2 – rater 3 = -0.070, rater 1 – rater 4 = -0.067, rater 2 – rater 4 = -0.138, rater 3 – rater 4 = -0.117, rater 1 – rater 5 = -0.105, rater 1 – rater 6 = -0.040, and rater 4 – rater 6 = -0.176) indicate that these raters have a poor level of agreement. The Cohen’s average kappa of -0.008 also suggests a poor level of agreement amongst the raters.

**Table 4:** Raters’ agreement reliability on traditional lecture methodology used in OSCE training

Cohen’s Unweighted Kappa				
			**95% CI**	
**Ratings**	**Unweighted Kappa**	**SE**	**Lower**	**Upper**
Average Kappa	**-0.008**			
Rater 1 - Rater 2	0.007	0.064	-0.119	0.133
Rater 1 - Rater 3	**-0.146**	0.072	-0.287	-0.005
Rater 2 - Rater 3	**-0.070**	0.055	-0.177	0.038
Rater 1 - Rater 4	**-0.067**	0.084	-0.232	0.098
Rater 2 - Rater 4	**-0.138**	0.099	-0.332	0.055
Rater 3 - Rater 4	**-0.117**	0.073	-0.261	0.027
Rater 1 - Rater 5	**-0.105**	0.070	-0.241	0.032
Rater 2 - Rater 5	0.231	0.132	-0.028	0.489
Rater 3 - Rater 5	0.039	0.091	-0.139	0.217
Rater 4 - Rater 5	0.115	0.155	-0.189	0.418
Rater 1 - Rater 6	**-0.040**	0.077	-0.190	0.110
Rater 2 - Rater 6	0.111	0.148	-0.178	0.401
Rater 3 - Rater 6	0.203	0.103	0.001	0.405
Rater 4 - Rater 6	**-0.176**	0.107	-0.385	0.034
Rater 5 - Rater 6	0.033	0.142	-0.244	0.311
***Fleiss’ Kappa***				
			**95% CI**	
**Ratings**	**Fleiss’ Kappa**	**SE**	**Lower**	**Upper**
Overall	**-0.025**	0.029	-0.082	0.031
4	**-0.080**	0.061	-0.199	0.039
5	**-0.080**	0.061	-0.210	0.028
6	**-0.080**	0.061	-0.199	0.039
7	**-0.013**	0.061	-0.132	0.106
8	**-0.031**	0.061	-0.150	0.088
9	0.065	0.061	-0.054	0.184
10	0.118	0.061	-0.001	0.237

Furthermore, the total agreement of the raters was poor, as indicated by the overall Fleiss’ kappa of -0.025. The Fleiss’ kappa values were also included in the rating system from 4 to 10 out of 10. Additionally, this reflects that the raters have poor agreement between ratings of 4, 5, 6, 7, and 8, with Fleiss’ kappa values of -0.080, -0.080, -0.080, -0.013, and − 0.031, respectively (see Table 3). According to Landis and Koch [30], Cohen’s kappa and Fleiss’ kappa results less than 0 indicate poor agreement.

Table 5 shows the rated performance of the OSCE examinees in the three instructional methodologies. It is evident that the examinees who were trained through the simulation methodology performed better, with an average mean of 9.01 out of 10 and a SD of 0.70 compared to online (mean = 5.39/10; SD = 0.50) and traditional (mean = 7.01/10; SD = 0.61) methodologies. Moreover, the p-value of 0.0000, which is below the significance level of 0.01, indicates a significant difference in the performance of the OSCE examinees in the three instructional methodologies. Furthermore, 11 (20.37) examinees who had undergone OSCE training through simulation were rated very good and seven (12.96) received good scores. All 18 examinees (33.33) who had undergone OSCE training through traditional lectures received good scores, whilst two (3.70) of the examinees who had undergone OSCE training through the online medium received good scores, and the majority of them 16 (29.63) received poor scores. Overall, 11 (20.37) of the total 54 examinees were rated as very good, 27 (50.00) were rated good, and 16 (29.63) were rated poor (see Table 4). Table 6 shows the post hoc analysis in comparing the rated performance of the OSCE examinees in the three instructional methodologies. The analysis revealed that the differences in the performance exist between all pairs of instructional methodologies, with a p-value of 0.000, which is less than the significance level of 0.01 (see Table 5).

**Table 5:** Rated performance of the OSCE examinees in the three instructional methodologies

Groups	Mean		Std. Dev.		ANOVA F-value			*P*-value	
Online	5.39		0.50		161.28			0.0000	
Simulation	9.01		0.70						
Traditional lecture	7.01		0.61						
		**F (%)**							
**Groups**		**Very Good**		**Good**		**Poor**	**Very Poor**		**Total**
Online		0 (0.00)		2 (3.70)		16 (29.63)	0 (0.00)		18 (33.33)
Simulation		11 (20.37)		7 (12.96)		0 (0.00)	0 (0.00)		18 (33.33)
Traditional lecture		0 (0.00)		18 (33.33)		0 (0.00)	0 (0.00)		18 (33.33)
**Total**		**11 (20.37)**		**27 (50.00)**		**16 (29.63)**	**0 (0.00)**		**54 (100)**

Table 7 presents the overall perceptions of raters, examinees, and trainer’s perception regarding the effectiveness of the three instructional methodologies used in OSCE training. In terms of online methodology, the rater’s perceptions have an overall mean average of 1.36, with a SD of 0.29. The examinee’s perception had an overall mean average of 1.33 with a SD of 0.45, whilst the trainers had an overall mean average of 1.17 with a SD of 0.00. Moreover, the raters, examinees, and trainers agreed that the online methodology in OSCE training was not effective.

For the simulation methodology, the rater’s perceptions had an overall mean average of 2.97 with a SD of 0.14. The examinee’s perception had an overall mean average of 3.00 with a SD of 0.00, whilst the trainers had an overall mean average of 2.84 with a SD of 0.24. Moreover, the raters, examinees, and trainers agreed that the simulation methodology in OSCE training was effective. Conversely, however, for the traditional lecture methodology, the rater’s perceptions had an overall mean average of 2.01, with a SD of 0.35. The examinee’s perception had an overall mean average of 2.00, with a SD of 0.44, whilst the trainers had an overall mean average of 1.83 with a SD of 0.24. Moreover, the raters, examinees, and trainers agreed that the simulation methodology in OSCE training was slightly effective.

Furthermore, the raters, examinees, and trainers on each instructional methodology consistently agreed on their perceptions, as supported by their responses, which included the following p-values: online = 0.739545, simulation = 0.31291, and traditional lecture = 0.808114. Indeed, all of these are greater than the significance level of 0.01, which indicates no significant difference (see Table 6).

**Table 6:** Post hoc analysis

Mean Difference (*p*-value)			
**Groups**	Online	Simulation	
Simulation	3.62 (0.000)		
Traditional lecture	1.62 (0.000)	-2.00 (0.000)	

**Table 7:** Agreement of raters, examinees, and trainers on the effectiveness of the instructional methodologies used in OSCE training

Online	Mean	SD	Verbal Interpretation	ANOVA F-value	*P*-value
Rater perception	1.36	0.29	Not effective	0.305713	0.739545
Examinee perception	1.33	0.45	Not effective		
Trainer perception	1.17	0.00	Not effective		
***Simulation***					
Rater perception	2.97	0.14	Effective		
Examinee perception	3.00	0.00	Effective	1.222558	0.31291
Trainer perception	2.83	0.24	Effective		
***Traditional Lecture***					
Rater perception	2.01	0.35	Slightly effective	0.215038	0.808114
Examinee perception	2.00	0.44	Slightly effective		
Trainer perception	1.83	0.24	Slightly effective		

Table 8 presents the comparison of the effectiveness of online, simulation, and traditional lecture based on the raters, examinees, and trainers’ perceptions. The simulation method had the highest mean value across all the methodologies, making it the most effective methodology. The raters had a mean value of 3.00 with a SD of 0.00, whilst the examinees had a mean value of 2.97 with a SD of 0.14, and the trainers had a mean value of 2.84 with a SD of 0.23, respectively. However, out of the three methodologies, the online method had the lowest mean value. Furthermore, the perceptions of the raters, examinees, and trainers regarding the three methodologies showed significant differences, supported by the p-value of 0.0000, which is below the significance level of 0.01 accordingly (see Table 7).

**Table 8:** Comparison of the effectiveness of online, simulation, and traditional lectures based on raters, examinees, and trainers’ perceptions

Raters				
**Groups**	**Mean**	**Std. Dev.**	**ANOVA F-value**	**P-value**
Online	1.33	0.45	32.09	0.0000
Simulation	3.00	0.00		
Traditional lecture	2.00	0.44		
***Examinees***				
Online	1.36	0.29	159.32	0.0000
Simulation	2.97	0.14		
Traditional lecture	2.01	0.35		
***Trainers***				
Online	1.17	0.00		0.0000
Simulation	2.84	0.23	38.70	
Traditional lecture	1.84	0.23		

Table 9 presents the post hoc analysis in comparing the effectiveness of three instructional methodologies used in OSCE training. For the raters, all of the p-values between each pair were less than the 0.01 level of significance. This implies that all three interventions significantly differ from each other according to the raters’ point of view. For the examinees, all of the p-values between each pair were less than the 0.01 level of significance. This implies that all three interventions significantly differ from each other from the examinees’ point of view. Furthermore, for the trainers, all of the p-values between each pair were less than the 0.05 level of significance, with the exception of those between online and traditional (p-value = 0.119). This implies that all the pairs of interventions significantly differ from each other from the point of view of the trainers, but not between online and traditional (see Table 8).

**Table 9:** Post hoc analysis

Mean Difference (*p*-value)			
Raters			
**Groups**	Online	Simulation	
Simulation	1.67 (0.000)		
Traditional lecture	0.67 (0.019)	-1 (0.001)	
***Examinees***			
**Cohorts**	Online	Simulation	
Simulation	1.61 (0.000)		
Traditional lecture	0.66 (0.000)	-0.96 (0.000)	
***Trainers***			
**Cohorts**	Online	Simulation	
Simulation	1.66 (0.009)		
Traditional lecture	0.67 (0.119)	-1 (0.040)	

## Discussion

This study evaluated the effectiveness of different instructional methodologies in preparing raters and examiners for the OSCE. The findings indicate that OSCE training through simulation is the most effective method compared with training through online and traditional methodologies. In terms of rater agreement reliability, raters who underwent OSCE training through simulation had a slight level of agreement compared with those who underwent OSCE training through online and traditional lectures and demonstrated a poor level of agreement. The performance of most examinees who underwent OSCE training through simulation was rated as very good. Furthermore, the perceptions of the raters, examinees, and trainers regarding the instructional methodology of their simulated OSCE training were consistent. However, those who participated in both online and traditional lectures reported that they were ineffective. Interestingly, the results for the agreement reliability of the raters; the performance of the examinees; and the perspectives of the raters, examinees, and trainers regarding the training methodologies conducted were congruent. Therefore, simulation was deemed the most effective methodology for training OSCE raters and examinees.

Assessing the competencies of nursing students through the OSCE is important for gauging the extent of their learning and identifying their strengths and weaknesses. However, the OSCE implementation only succeeds after proper training of raters and the comprehensive orientation of the examinees. Furthermore, simulations can supplement or even replace traditional lectures and online teaching platforms for training raters and examinees in the OSCE.

The National League for Nursing (NLN) recommends integrating simulations into nursing curricula to cultivate a clear line of sight between the in-depth learning of students and successful learning outcomes [31]. Moreover, Guerrero [4] suggested that well-designed and structured simulation training can effectively replace up to 40% of the traditional instructional methods in nursing education. This aligns with the NLN’s emphasis on fostering immersive and observational learning experiences through simulations [4, 31]. OSCEs are forms of simulation that can be formative but are mostly summative. However, a structured and meticulous design is required to ensure optimal practices and assessments [32].

A comprehensive and accurate assessment of the knowledge, skills, and attitudes of nursing students plays a pivotal role in fostering their professional development and preparing them for clinical practice [33, 34]. To ensure an objective assessment, the raters rely on standardized rating scales or checklists. These tools guide students’ observations of their performance, enabling consistent judgment of their mastery of key competencies [33, 35]. However, Hyde et al. [5] reported that less experienced OSCE raters were more likely to judge more stringently than more experienced examiners.

In the study of Khamisa et al. [36], a comparison of the effects of online and traditional in-person (face-to-face) orientation of OSCE raters revealed that both approaches yielded the same outcome. This result is similar to the findings of the present study, i.e. that the online and traditional methodologies of training raters for the OSCE are similar but imply a poor level of agreement. Moreover, Sigalet et al. [37] reported that, during their OSCE, raters training through simulation utilising standardized learners and low-fidelity simulators, the OSCE raters had a moderate agreement across 42 OSCE training scenarios. The result is comparable to the outcome of those OSCE raters who were trained through simulation in the present study, who exhibited a slight level of agreement compared to those trained online and via the traditional in-person (face-to-face) method. However, it is important to be aware that all assessment methods have flaws, limitations, and shortcomings [5]. To reduce sources of measurement error and ensure the examinees’ confidence that their scores accurately reflect their performance, formal training of raters regarding OSCE organisation and implementation is essential [37, 38], since OSCE raters with less experience were more likely to judge compared to those raters with a wealth of experience [5]. Moreover, it is also important that students are able to undergo appropriate OSCE training and preparation first to ensure that they are familiar with the process [7]. The OSCE experience of nursing students allows them to easily adapt in the actual clinical scenario and gain more confidence in their performance in the real world of clinical practice [39].

Additionally, a systematic review conducted by Kassabry [7] reported that the OSCE increases the knowledge, self-confidence, satisfaction, and clinical judgment acquisition of nursing students in comparison to other traditional evaluation tools. However, teachers must ensure that students are familiar with, and undergo, appropriate training for the OSCE to ensure that the method is followed accordingly. Furthermore, 67% (12 of 18) of studies reported that well-structured and sequenced OSCE with sufficient instructions provided excellent assessment reliability [40–42].

Building on the research by Lee et al. [25] demonstrating that SBL is a valuable tool for learning advancement, this study explored the potential of SBL to enhance the contextual learning experiences of students [4, 33]. SBL has shown promising outcomes in numerous studies, yielding exciting results for learners in various fields [26]. However, further research is necessary to discover the full spectrum of its efficacy and identify the specific types of performance most affected by SBL interventions [4].

### Limitations

This study had certain limitations. First, it was conducted at only one higher education institution in Saudi Arabia. Second, most participants were women. Third, the number of participating raters, examinees, and trainers was small, and the backgrounds of the raters and examinees were homogenous. Particularly, there were only two well-trained OSCE specialists who were invited to participate, and who also served as evaluators. Moreover, the effect size for the t-tests was not calculated because the p-values had already been provided for both practical and statistical significance. Further research must be conducted in multiple settings and locations to confirm the effectiveness of the simulation in training raters and examinees on the OSCE.

## Conclusions

This study provides insights into the importance of selecting an appropriate assessment method to evaluate the performance and competencies of nursing students. Many academic institutions worldwide have adopted the OSCE to assess clinical competency. However, to achieve a positive and meaningful outcome, raters and examinees should be appropriately trained, and selecting the best instructional methodology through which to train them is crucial. The findings of this study suggest that simulation is the most effective methodology for training raters and examinees in the OSCE. It can supplement or replace traditional OSCE lectures and online teaching platforms.

### Electronic supplementary material

Below is the link to the electronic supplementary material.

Supplementary Material 1

Supplementary Material 2

## Data Availability

The data supporting the findings of this study are available from the corresponding author upon reasonable request.

## Abbreviations

OSCE: Objective Structured Clinical Examination
SBL: Simulation-Based Learning
BSN: Bachelor of Science in Nursing
SD: Standard Deviations
NLN: National League for Nursing
