# Supplementary material for: Raters and examinees training for objective structured clinical examination: comparing the effectiveness of three instructional methodologies
Source: BMC Nurs. 2024 Jul 23;23:500. doi: 10.1186/s12912-024-02183-6 (PMC11265468; doi:10.1186/s12912-024-02183-6)
Supplement: Supplementary file 1 — Supplementary Material 1 [file 12912_2024_2183_MOESM1_ESM.docx]

**Trainers, Raters, and Examinees Feedback with the OSCE Orientation/Training**

**Trainers *(Observers)* Feedback**

| **No.** | **Feedback** | **Agree**  **(3)** | **Partially Agree**  **(2)** | **Disagree**  **(1)** |
| --- | --- | --- | --- | --- |
| 1 | The goals and objectives of the OSCE orientation/training (*pilot test*) is achieved. |  |  |  |
| 2 | The examinees followed the OSCE instructions very well. |  |  |  |
| 3 | The OSCE raters followed their tasks accordingly. |  |  |  |
| 4 | The OSCE organizer and timekeeper followed their task accordingly. |  |  |  |
| 5 | The recommended time of each cycle was followed accordingly. |  |  |  |
| 6 | The overall conduct of the OSCE was organized. |  |  |  |

**Raters Feedback**

| **No.** | **Feedback** | **Agree**  **(3)** | **Partially Agree**  **(2)** | **Disagree**  **(1)** |
| --- | --- | --- | --- | --- |
| 1 | The channel/method of delivering OSCE orientation/training is effective and efficient. |  |  |  |
| 2 | I felt prepared rating the examinees during summative OSCE. |  |  |  |
| 3 | I felt more confidence in rating the examinees during summative OSCE. |  |  |  |
| 4 | I felt less anxious and stress rating the examinees after the orientation. |  |  |  |
| 5 | The overall goals and objectives of the OSCE orientation/training is achieved. |  |  |  |

**Examinees Feedback**

| **No.** | **Feedback** | **Agree**  **(3)** | **Partially Agree**  **(2)** | **Disagree**  **(1)** |
| --- | --- | --- | --- | --- |
| 1 | The channel/method of delivering OSCE orientation is effective and efficient. |  |  |  |
| 2 | I felt prepared for the summative OSCE. |  |  |  |
| 3 | I felt more confidence in taking the summative OSCE. |  |  |  |
| 4 | I felt less anxious and stress going into summative OSCE after the orientation. |  |  |  |
| 5 | The overall goals and objectives of the OSCE orientation/training is achieved. |  |  |  |
