# Supplementary material for: Raters and examinees training for objective structured clinical examination: comparing the effectiveness of three instructional methodologies
Source: BMC Nurs. 2024 Jul 23;23:500. doi: 10.1186/s12912-024-02183-6 (PMC11265468; doi:10.1186/s12912-024-02183-6)
Supplement: Supplementary file 2 — Supplementary Material 2 [file 12912_2024_2183_MOESM2_ESM.docx]

**Station 1**

**Examiner’s Checklist**

| **No** | **Procedure** | **Performed Correctly**  **(3)** | **Performed Incompletely**  **(2)** | **Performed Incorrectly (1)** | **Not Performed**  **(0)** |  |
| --- | --- | --- | --- | --- | --- | --- |
|  | Gather equipment. Check each medication order against the original order in the medical record according to facility policy. Clarify any inconsistencies. Check the patient’s chart for allergies. |  |  |  |  |  |
|  | Know the actions, special nursing considerations, safe dose ranges, purpose of administration, and adverse effects of the medications to be administered. Consider the appropriateness of the medication for this patient. |  |  |  |  |  |
|  | Perform hand hygiene. |  |  |  |  |  |
|  | Move the medication cart to the outside of the patient’s room or prepare for administration in the medication area. |  |  |  |  |  |
|  | Unlock the medication cart or drawer. Enter pass code and scan employee identification, if required. |  |  |  |  |  |
|  | Prepare medications for one patient at a time. |  |  |  |  |  |
|  | Read the MAR and select the proper medication from the patient’s medication drawer or unit stock. |  |  |  |  |  |
|  | Compare the label with the MAR. Check expiration dates and perform calculations, if necessary. |  |  |  |  |  |
|  | If necessary, withdraw medication from an ampule or vial. |  |  |  |  |  |
|  | When all medications for one patient have been prepared, recheck the label with the MAR before taking the medications to the patient. |  |  |  |  |  |
|  | Lock the medication cart before leaving it. |  |  |  |  |  |
|  | Transport medications to the patient’s bedside carefully and keep the medications in sight at all times. |  |  |  |  |  |
|  | Ensure that the patient receives the medications at the correct time. |  |  |  |  |  |
|  | Perform hand hygiene and put on PPE, if indicated. |  |  |  |  |  |
|  | Identify the patient. Usually, the patient should be identified using two methods. Compare information with the MAR.   - 1. Check the name and identification number on the patient’s identification band.   2. Ask the patient to state his or her name and birth date, based on facility policy.   3. If the patient cannot identify him- or herself, verify the patient’s identification with a staff member who knows the patient for the second source. |  |  |  |  |  |
|  | Close the door to the room or pull the bedside curtain. |  |  |  |  |  |
|  | Complete necessary assessments before administering medications. Check allergy bracelet or ask the patient about allergies. Explain the purpose and action of the medication to the patient. |  |  |  |  |  |
|  | Put on clean gloves. |  |  |  |  |  |
|  | Select an appropriate administration site. Assist the patient to the appropriate position for the site chosen. Drape as needed to expose only area of site to be used. |  |  |  |  |  |
|  | Cleanse the site with an antimicrobial swab while wiping with a firm, circular motion and moving outward from the injection site. Allow the skin to dry. |  |  |  |  |  |
|  | Remove the needle cap with the nondominant hand by pulling it straight off. |  |  |  |  |  |
|  | Use the nondominant hand to spread the skin taut over the injection site. |  |  |  |  |  |
|  | Hold the syringe in the dominant hand, between the thumb and forefinger with the bevel of the needle up. |  |  |  |  |  |
|  | Hold the syringe at a 5- to 15-degree angle from the site. Place the needle almost flat against the patient’s skin, bevel side up, and insert the needle into the skin. Insert the needle only about 1⁄8 inch with entire bevel under the skin. |  |  |  |  |  |
|  | Once the needle is in place, steady the lower end of the syringe. Slide your dominant hand to the end of the plunger. |  |  |  |  |  |
|  | Slowly inject the agent while watching for a small wheal or blister to appear. |  |  |  |  |  |
|  | Withdraw the needle quickly at the same angle that it was inserted. Do not recap the used needle. Engage the safety shield or needle guard. |  |  |  |  |  |
|  | Do not massage the area after removing needle. Tell patient not to rub or scratch the site. If necessary, gently blot the site with a dry gauze square. Do not apply pressure or rub the site. |  |  |  |  |  |
|  | Assist the patient to a position of comfort. |  |  |  |  |  |
|  | Discard the needle and syringe in the appropriate receptacle. |  |  |  |  |  |
|  | Remove gloves and additional PPE, if used. Perform hand hygiene. |  |  |  |  |  |
|  | Document the administration of the medication immediately after administration. |  |  |  |  |  |
|  | Evaluate the patient’s response to medication within appropriate time frame. |  |  |  |  |  |
|  | Observe the area for signs of a reaction at determined intervals after administration. Inform the patient of the need for inspection. |  |  |  |  |  |
| **Total Raw Score ……. /102** | | | | | | |
| **Final Score ……. /10** | | | | | | |

**Station 2**

**Examiner’s Checklist**

| **No** | **Procedure** | **Performed Correctly**  **(3)** | **Performed Incompletely**  **(2)** | **Performed Incorrectly (1)** | **Not Performed**  **(0)** |  |
| --- | --- | --- | --- | --- | --- | --- |
|  | Gather equipment. Check each medication order against the original order in the medical record according to facility policy. Clarify any inconsistencies. Check the patient’s chart for allergies. |  |  |  |  |  |
|  | Know the actions, special nursing considerations, safe dose ranges, purpose of administration, and adverse effects of the medications to be administered. Consider the appropriateness of the medication for this patient. |  |  |  |  |  |
|  | Perform hand hygiene. |  |  |  |  |  |
|  | Move the medication cart to the outside of the patient’s room or prepare for administration in the medication area. |  |  |  |  |  |
|  | Unlock the medication cart or drawer. Enter pass code and scan employee identification, if required. |  |  |  |  |  |
|  | Prepare medications for one patient at a time. |  |  |  |  |  |
|  | Read the MAR and select the proper medication from the patient’s medication drawer or unit stock. |  |  |  |  |  |
|  | Compare the label with the MAR. Check expiration dates and perform calculations, if necessary. |  |  |  |  |  |
|  | If necessary, withdraw medication from an ampule or vial. |  |  |  |  |  |
|  | When all medications for one patient have been prepared, recheck the label with the MAR before taking the medications to the patient. |  |  |  |  |  |
|  | Lock the medication cart before leaving it. |  |  |  |  |  |
|  | Transport medications to the patient’s bedside carefully and keep the medications in sight at all times. |  |  |  |  |  |
|  | Ensure that the patient receives the medications at the correct time. |  |  |  |  |  |
|  | Perform hand hygiene and put on PPE, if indicated. |  |  |  |  |  |
|  | Identify the patient. Usually, the patient should be identified using two methods. Compare information with the MAR.   - 1. Check the name and identification number on the patient’s identification band.   2. Ask the patient to state his or her name and birth date, based on facility policy.   3. If the patient cannot identify him- or herself, verify the patient’s identification with a staff member who knows the patient for the second source. |  |  |  |  |  |
|  | Close the door to the room or pull the bedside curtain. |  |  |  |  |  |
|  | Complete necessary assessments before administering medications. Check allergy bracelet or ask the patient about allergies. Explain the purpose and action of the medication to the patient. |  |  |  |  |  |
|  | Put on clean gloves. |  |  |  |  |  |
|  | Select an appropriate administration site. |  |  |  |  |  |
|  | Assist the patient to the appropriate position for the site chosen. Drape, as needed, to expose only the area of site being used. |  |  |  |  |  |
|  | Identify the appropriate landmarks for the site chosen. |  |  |  |  |  |
|  | Cleanse the area around the injection site with an antimicrobial swab. Use a firm, circular motion while moving outward from the injection site. Allow area to dry. |  |  |  |  |  |
|  | Remove the needle cap by pulling it straight off. Hold the syringe in your dominant hand between the thumb and forefinger. |  |  |  |  |  |
|  | Displace the skin in a Z-track manner by pulling the skin down or to one side about 1 inch (2.5 cm) with your nondominant hand and hold the skin and tissue in this position. |  |  |  |  |  |
|  | Quickly dart the needle into the tissue so that the needle is perpendicular to the patient’s body. This should ensure that it is given using an angle of injection between 72 and 90 degrees. |  |  |  |  |  |
|  | As soon as the needle is in place, use the thumb and forefinger of your nondominant hand to hold the lower end of the syringe. Slide your dominant hand to the end of the plunger. Inject the solution slowly (10 sec/mL of medication). |  |  |  |  |  |
|  | Once the medication has been instilled, wait 10 seconds before withdrawing the needle. |  |  |  |  |  |
|  | Withdraw the needle smoothly and steadily at the same angle at which it was inserted, supporting tissue around the injection site with your nondominant hand. |  |  |  |  |  |
|  | Apply gentle pressure at the site with a dry gauze. Do not massage the site. |  |  |  |  |  |
|  | Do not recap the used needle. Engage the safety shield or needle guard, if present. Discard the needle and syringe in the appropriate receptacle. |  |  |  |  |  |
|  | Assist the patient to a position of comfort. |  |  |  |  |  |
|  | Remove gloves and additional PPE, if used. Perform hand hygiene. |  |  |  |  |  |
|  | Document the administration of the medication immediately after administration. |  |  |  |  |  |
|  | Evaluate the patient’s response to medication within an appropriate time frame. Assess site, if possible, within 2 to 4 hours after administration. |  |  |  |  |  |
| **Total Raw Score ……. /102** | | | | | | |
| **Final Score ……. /10** | | | | | | |

**Station 3**

**Examiner’s Checklist**

| **No** | **Procedure** | **Performed Correctly**  **(3)** | **Performed Incompletely**  **(2)** | **Performed Incorrectly (1)** | **Not Performed**  **(0)** |  |
| --- | --- | --- | --- | --- | --- | --- |
|  | Verify the medical order for insertion of an NG tube. |  |  |  |  |  |
|  | Perform hand hygiene and put on PPE, if indicated. |  |  |  |  |  |
|  | Identify the patient. |  |  |  |  |  |
|  | Explain the procedure to the patient and provide the rationale as to why the tube is needed. Discuss the associated discomforts that may be experienced and possible interventions that may allay this discomfort. Answer any questions as needed. |  |  |  |  |  |
|  | Gather equipment, including selection of the appropriate NG tube. |  |  |  |  |  |
|  | Close the patient’s bedside curtain or door. Raise bed to a comfortable working position. Assist the patient to high Fowler’s position or elevate the head of the bed 45 degrees if the patient is unable to maintain upright position. Drape chest with bath towel or disposable pad. Have emesis basin and tissues handy. |  |  |  |  |  |
|  | Measure the distance to insert tube by placing tip of tube at patient’s nostril and extending to tip of earlobe and then to tip of xiphoid process. Mark tube with an indelible marker. |  |  |  |  |  |
|  | Put on gloves. Lubricate tip of tube (at least 2”–4”) with water-soluble lubricant. Apply topical anesthetic to nostril and oropharynx, as appropriate. |  |  |  |  |  |
|  | After selecting the appropriate nostril, ask patient to slightly flex head back against the pillow. Gently insert the tube into the nostril while directing the tube upward and backward along the floor of the nose. Patient may gag when tube reaches pharynx. Provide tissues for tearing or watering of eyes. Offer comfort and reassurance to the patient. |  |  |  |  |  |
|  | When pharynx is reached, instruct patient to touch chin to chest. Encourage patient to sip water through a straw or swallow even if no fluids are permitted. Advance tube in downward and backward direction when patient swallows. Stop when patient breathes. If gagging and coughing persist, stop advancing the tube and check placement of tube with tongue blade and flashlight. If tube is curled, straighten the tube and attempt to advance again. Keep advancing tube until pen marking is reached. Do not use force. Rotate tube if it meets resistance. |  |  |  |  |  |
|  | Discontinue procedure and remove tube if there are signs of distress, such as gasping, coughing, cyanosis, and inability to speak or hum. |  |  |  |  |  |
|  | Secure the tube loosely to the nose or cheek until it is determined that the tube is in the patient’s stomach:   - 1. Attach syringe to end of tube and aspirate a small amount of stomach contents.   2. Measure the pH of aspirated fluid using pH paper or a meter. Place a drop of gastric secretions onto pH paper or place small amount in plastic cup and dip the pH paper into it. Within 30 seconds, compare the color on the paper with the chart supplied by the manufacturer.   3. Visualize aspirated contents, checking for color and consistency.   4. Obtain radiograph (x-ray) of placement of tube, based on facility policy (and ordered by physician). |  |  |  |  |  |
|  | Apply skin barrier to tip and end of nose and allow to dry. Remove gloves and secure tube with a commercially prepared device (follow manufacturer’s directions) or tape to patient’s nose. To secure with tape:   - 1. Cut a 4” piece of tape and split bottom 2” or use pack- aged nose tape for NG tubes.   2. Place unsplit end over bridge of patient’s nose.   3. Wrap split ends under tubing and up and over onto nose. Be careful not to pull tube too tightly against nose. |  |  |  |  |  |
|  | Put on gloves. Clamp tube and remove the syringe. Cap the tube or attach tube to suction according to the medical order. |  |  |  |  |  |
|  | Measure length of exposed tube. Reinforce marking on tube at nostril with indelible ink. Ask the patient to turn their head to the side opposite the nostril the tube is inserted. Secure tube to patient’s gown by using rubber band or tape and safety pin. For additional support, tube can be taped onto patient’s cheek using a piece of tape. If a double-lumen tube (e.g., Salem sump) is used, secure vent above stomach level. Attach at shoulder level. |  |  |  |  |  |
|  | Assist with or provide oral hygiene at 2- to 4-hour intervals. Lubricate the lips generously and clean nares and lubricate as needed. Offer analgesic throat lozenges or anesthetic spray for throat irritation if needed. |  |  |  |  |  |
|  | Remove equipment and return patient to a position of comfort. Remove gloves. Raise side rail and lower bed. |  |  |  |  |  |
|  | Remove additional PPE, if used. Perform hand hygiene. |  |  |  |  |  |
| **Total Raw Score ……. /54** | | | | | | |
| **Final Score ……. /10** | | | | | | |

**Station 4**

**Examiner’s Checklist**

| **No** | **Procedure** | **Performed Correctly**  **(3)** | **Performed Incompletely**  **(2)** | **Performed Incorrectly (1)** | **Not Performed**  **(0)** |  |
| --- | --- | --- | --- | --- | --- | --- |
|  | Review chart for any limitations in physical activity. Confirm the medical order for indwelling catheter insertion. |  |  |  |  |  |
|  | Bring catheter kit and other necessary equipment to the bedside. Obtain assistance from another staff member, if necessary. |  |  |  |  |  |
|  | Perform hand hygiene and put on PPE, if indicated. |  |  |  |  |  |
|  | Identify the patient. |  |  |  |  |  |
|  | Close curtains around bed and close the door to the room, if possible. Discuss the procedure with the patient and assess patient’s ability to assist with the procedure. Ask the patient if he has any allergies, especially to latex or iodine. |  |  |  |  |  |
|  | Provide good lighting. Artificial light is recommended (use of a flashlight requires an assistant to hold and position it). Place a trash receptacle within easy reach. |  |  |  |  |  |
|  | Adjust the bed to a comfortable working height. Stand on the patient’s right side if you are right-handed, patient’s left side if you are left-handed. |  |  |  |  |  |
|  | Position the patient on his back with thighs slightly apart. Drape the patient so that only the area around the penis is exposed. Slide waterproof pad under patient. |  |  |  |  |  |
|  | Put on clean gloves. Clean the genital area with washcloth, skin cleanser, and warm water. Clean the tip of the penis first, moving the washcloth in a circular motion from the meatus outward. Wash the shaft of the penis using downward strokes toward the pubic area. Rinse and dry. Remove gloves. Perform hand hygiene again. |  |  |  |  |  |
|  | Prepare urine drainage setup if a separate urine collection system is to be used. Secure to bed frame according to manufacturer’s directions. |  |  |  |  |  |
|  | Open sterile catheterization tray on a clean overbed table, using sterile technique. |  |  |  |  |  |
|  | Put on sterile gloves. Open sterile drape and place on patient’s thighs. Place fenestrated drape with opening over penis. |  |  |  |  |  |
|  | Place catheter set on or next to patient’s legs on sterile drape. |  |  |  |  |  |
|  | Open all the supplies. Fluff cotton balls in tray before pouring antiseptic solution over them. Alternately, open package of antiseptic swabs. Open specimen container if specimen is to be obtained. Remove cap from syringe pre-filled with lubricant. |  |  |  |  |  |
|  | Place drainage end of catheter in receptacle. If the catheter is pre-attached to sterile tubing and drainage container (closed drainage system), position catheter and setup within easy reach on sterile field. Ensure that clamp on drainage bag is closed. |  |  |  |  |  |
|  | Lift penis with nondominant hand. Retract foreskin in uncircumcised patient. Be prepared to keep this hand in this position until catheter is inserted, and urine is flowing well and continuously. Using the dominant hand and the forceps, pick up a cotton ball or antiseptic swab. Using a circular motion, clean the penis, moving from the meatus down the glans of the penis. Repeat this cleansing motion two more times, using a new cotton ball/swab each time. Discard each cotton ball/swab after one use. |  |  |  |  |  |
|  | Hold penis with slight upward tension and perpendicular to patient’s body. Use the dominant hand to pick up the lubricant syringe. Gently insert tip of syringe with lubricant into urethra and instill the 10 mL of lubricant. |  |  |  |  |  |
|  | Use the dominant hand to pick up the catheter and hold it an inch or two from the tip. Ask the patient to bear down as if voiding. Insert catheter tip into meatus. Ask the patient to take deep breaths. Advance the catheter to the bifurcation or “Y” level of the ports. Do not use force to introduce the catheter. If the catheter resists entry, ask patient to breathe deeply and rotate catheter slightly. |  |  |  |  |  |
|  | Hold the catheter securely at the meatus with your nondominant hand. Use your dominant hand to inflate the catheter balloon. Inject the entire volume of sterile water supplied in the prefilled syringe. Once the balloon is inflated, the catheter may be gently pulled back into place. Replace foreskin over catheter. Lower penis. |  |  |  |  |  |
|  | Pull gently on catheter after balloon is inflated to feel resistance. |  |  |  |  |  |
|  | Attach catheter to drainage system, if necessary. |  |  |  |  |  |
|  | Remove equipment and dispose of it according to facility policy. Discard syringe in sharps container. Wash and dry  the perineal area as needed. |  |  |  |  |  |
|  | Remove gloves. Secure catheter tubing to the patient’s inner thigh or lower abdomen (with the penis directed toward the patient’s chest) with Velcro leg strap or tape. Leave some slack in catheter for leg movement. |  |  |  |  |  |
|  | Assist the patient to a comfortable position. Cover the patient with bed linens. Place the bed in the lowest position. |  |  |  |  |  |
|  | Secure drainage bag below the level of the bladder. Check that drainage tubing is not kinked, and that movement of side rails does not interfere with catheter or drainage bag. |  |  |  |  |  |
|  | Put on clean gloves. Obtain urine specimen immediately, if needed, from drainage bag. Label specimen. Send urine specimen to the laboratory promptly or refrigerate it. |  |  |  |  |  |
|  | Remove gloves and additional PPE, if used. Perform hand hygiene. |  |  |  |  |  |
| **Total Raw Score ……. /81** | | | | | | |
| **Final Score ……. /10** | | | | | | |

**Station 5**

**Examiner’s Checklist**

| **No** | **Procedure** | **Performed Correctly**  **(3)** | **Performed Incompletely**  **(2)** | **Performed Incorrectly (1)** | **Not Performed**  **(0)** |  |
| --- | --- | --- | --- | --- | --- | --- |
|  | Review chart for any limitations in physical activity. Confirm the medical order for indwelling catheter insertion. |  |  |  |  |  |
|  | Bring the catheter kit and other necessary equipment to the bedside. Obtain assistance from another staff member, if necessary. |  |  |  |  |  |
|  | Perform hand hygiene and put on PPE, if indicated. |  |  |  |  |  |
|  | Identify the patient. |  |  |  |  |  |
|  | Close curtains around bed and close the door to the room, if possible. Discuss the procedure with the patient and assess the patient’s ability to assist with the procedure. Ask the patient if she has any allergies, especially to latex or iodine. |  |  |  |  |  |
|  | Provide good lighting. Artificial light is recommended (use of a flashlight requires an assistant to hold and position it). Place a trash receptacle within easy reach. |  |  |  |  |  |
|  | Adjust the bed to a comfortable working height. Stand on the patient’s right side if you are right-handed, patient’s left side if you are left-handed. |  |  |  |  |  |
|  | Assist the patient to a dorsal recumbent position with knees flexed, feet about 2 feet apart, with her legs abducted. Drape patient. Alternately, the Sims’, or lateral, position can be used. Place the patient’s buttocks near the edge of the bed with her shoulders at the opposite edge and her knees drawn toward her chest. Allow the patient to lie on either side, depending on which position is easiest for the nurse and best for the patient’s comfort. Slide waterproof pad under patient. |  |  |  |  |  |
|  | Put on clean gloves. Clean the perineal area with washcloth, skin cleanser, and warm water, using a different corner of the washcloth with each stroke. Wipe from above orifice downward toward sacrum (front to back). Rinse and dry. Remove gloves. Perform hand hygiene again. |  |  |  |  |  |
|  | Prepare urine drainage setup if a separate urine collection system is to be used. Secure to bed frame according to manufacturer’s directions. |  |  |  |  |  |
|  | Open sterile catheterization tray on a clean overbed table using sterile technique. |  |  |  |  |  |
|  | Put on sterile gloves. Grasp upper corners of drape and unfold drape without touching unsterile areas. Fold back a corner on each side to make a cuff over gloved hands. Ask patient to lift her buttocks and slide sterile drape under her with gloves protected by cuff. |  |  |  |  |  |
|  | Based on facility policy, position the fenestrated sterile drape. Place a fenestrated sterile drape over the perineal area, exposing the labia. |  |  |  |  |  |
|  | Place sterile tray on drape between patient’s thighs. |  |  |  |  |  |
|  | Open all the supplies. Fluff cotton balls in tray before pouring antiseptic solution over them. Alternately, open package of antiseptic swabs. Open specimen container if specimen is to be obtained. |  |  |  |  |  |
|  | Lubricate 1 to 2 inches of catheter tip. |  |  |  |  |  |
|  | With thumb and one finger of nondominant hand, spread labia and identify meatus. Be prepared to maintain separation of labia with one hand until catheter is inserted, and urine is flowing well and continuously. If the patient is in the side-lying position, lift the upper buttock and labia to expose the urinary meatus. |  |  |  |  |  |
|  | Use the dominant hand to pick up a cotton ball or antiseptic swab. Clean one labial fold, top to bottom (from above the meatus down toward the rectum), then discard the cotton ball. Using a new cotton ball/swab for each stroke, continue to clean the other labial fold, then directly over the meatus. |  |  |  |  |  |
|  | With your uncontaminated, dominant hand, place the drainage end of the catheter in receptacle. If the catheter is pre-attached to sterile tubing and drainage container (closed drainage system), position catheter and setup within easy reach on sterile field. Ensure that clamp on drainage bag is closed. |  |  |  |  |  |
|  | Using your dominant hand, hold the catheter 2 to 3 inches from the tip and insert slowly into the urethra. Advance the catheter until there is a return of urine (approximately 2 to 3 inches [4.8 to 7.2 cm]). Once urine drains, advance catheter another 2 to 3 inches (4.8 to 7.2 cm). Do not force catheter through urethra into bladder. Ask patient to breathe deeply, and rotate catheter gently if slight resistance is met as catheter reaches external sphincter. |  |  |  |  |  |
|  | Hold the catheter securely at the meatus with your nondominant hand. Use your dominant hand to inflate the catheter balloon. Inject entire volume of sterile water supplied in prefilled syringe. |  |  |  |  |  |
|  | Pull gently on catheter after balloon is inflated to feel resistance. |  |  |  |  |  |
|  | Attach catheter to drainage system if not already pre-attached. |  |  |  |  |  |
|  | Remove equipment and dispose of it according to facility policy. Discard syringe in sharps container. Wash and dry the perineal area, as needed. |  |  |  |  |  |
|  | Remove gloves. Secure catheter tubing to the patient’s inner thigh with Velcro leg strap or tape. Leave some slack in catheter for leg movement. |  |  |  |  |  |
|  | Assist the patient to a comfortable position. Cover the patient with bed linens. Place the bed in the lowest position. |  |  |  |  |  |
|  | Secure drainage bag below the level of the bladder. Check that drainage tubing is not kinked, and that movement of side rails does not interfere with catheter or drainage bag. |  |  |  |  |  |
|  | Put on clean gloves. Obtain urine specimen immediately, if needed, from drainage bag. Label specimen. Send urine specimen to the laboratory promptly or refrigerate it. |  |  |  |  |  |
|  | Remove gloves and additional PPE, if used. Perform hand hygiene. |  |  |  |  |  |
| **Total Raw Score ……. /87** | | | | | | |
| **Final Score ……. /10** | | | | | | |

**Station 6**

**Examiner’s Checklist**

| **No** | **Procedure** | **Performed Correctly**  **(3)** | **Performed Incompletely**  **(2)** | **Performed Incorrectly (1)** | **Not Performed**  **(0)** |
| --- | --- | --- | --- | --- | --- |
|  | Check physician’s order or nursing care plan for frequency of blood pressure measurement. More frequent measurement may be appropriate based on nursing judgment. |  |  |  |  |
|  | Perform hand hygiene and put on PPE, if indicated. |  |  |  |  |
|  | Identify the patient. |  |  |  |  |
|  | Close curtains around bed and close the door to the room, if possible. Discuss procedure with patient and assess patient’s ability to assist with the procedure. Validate that the patient has relaxed for several minutes. |  |  |  |  |
|  | Put on gloves, if appropriate or indicated. |  |  |  |  |
|  | Select the appropriate arm for application of the cuff. |  |  |  |  |
|  | Have the patient assume a comfortable lying or sitting position with the forearm supported at the level of the heart and the palm of the hand upward. If the measurement is taken in the supine position, support the arm with a pillow. In the sitting position, support the arm yourself or by using the bedside table. If the patient is sitting, have the patient sit back in the chair so that the chair supports his or her back. In addition, make sure the patient keeps the legs uncrossed. |  |  |  |  |
|  | Expose the brachial artery by removing garments, or move a sleeve, if it is not too tight, above the area where the cuff will be placed. |  |  |  |  |
|  | Palpate the location of the brachial artery. Center the bladder of the cuff over the brachial artery, about midway on the arm, so that the lower edge of the cuff is about 2.5 to 5 cm (1 to 2 inches) above the inner aspect of the elbow. Line the artery marking on the cuff up with the patient’s brachial artery. The tubing should extend from the edge of the cuff nearer the patient’s elbow. |  |  |  |  |
|  | Wrap the cuff around the arm smoothly and snugly and fasten it. Do not allow any clothing to interfere with the proper placement of the cuff. |  |  |  |  |
|  | Check that the needle on the aneroid gauge is within the zero mark. If using a mercury manometer, check to see that the manometer is in the vertical position and that the mercury is within the zero level with the gauge at eye level. |  |  |  |  |
|  | Palpate the pulse at the brachial or radial artery by pressing gently with the fingertips. |  |  |  |  |
|  | Tighten the screw valve on the air pump. |  |  |  |  |
|  | Inflate the cuff while continuing to palpate the artery. Note the point on the gauge where the pulse disappears. |  |  |  |  |
|  | Deflate the cuff and wait 1 minute. |  |  |  |  |
|  | Assume a position that is no more than 3 feet away from the gauge. |  |  |  |  |
|  | Place the stethoscope earpieces in your ears. Direct the earpieces forward into the canal and not against the ear itself. |  |  |  |  |
|  | Place the bell or diaphragm of the stethoscope firmly but with as little pressure as possible over the brachial artery. Do not allow the stethoscope to touch clothing or the cuff. |  |  |  |  |
|  | Pump the pressure 30 mm Hg above the point at which the systolic pressure was palpated and estimated. Open the valve on the manometer and allow air to escape slowly (allowing the gauge to drop 2 to 3 mm per second). |  |  |  |  |
|  | Note the point on the gauge at which the first faint, but clear, sound appears that slowly increases in intensity. Note this number as the systolic pressure. Read the pressure to the closest 2 mm Hg. |  |  |  |  |
|  | Do not reinflate the cuff once the air is being released to recheck the systolic pressure reading. |  |  |  |  |
|  | Note the point at which the sound completely disappears. |  |  |  |  |
|  | Allow the remaining air to escape quickly. Repeat any suspicious reading but wait at least 1 minute. Deflate the cuff completely between attempts to check the blood pressure. |  |  |  |  |
|  | When measurement is completed, remove the cuff. Remove gloves, if worn. Cover the patient and help him or her to a position of comfort. |  |  |  |  |
|  | Remove additional PPE, if used. Perform hand hygiene. |  |  |  |  |
|  | Clean the diaphragm of the stethoscope with the alcohol wipe. Clean and store the sphygmomanometer. |  |  |  |  |
| **Total Raw Score ……. /78** | | | | | |
| **Final Score ……. /10** | | | | | |
